# Immunohistochemical Distribution of Serotonin Transporter (SERT) in the Optic Lobe of the Honeybee, *Apis mellifera*

**DOI:** 10.3390/ani12162032

**Published:** 2022-08-10

**Authors:** Cristiano Bombardi, Giulia Salamanca, Claudio Tagliavia, Annamaria Grandis, Fanny Mille, Maria Grazia De Iorio, Giulietta Minozzi

**Affiliations:** 1 Department of Veterinary Medical Sciences, University of Bologna, Via Tolara di Sopra, 50, Ozzano dell’Emilia, 40064 Bologna, Italy; 2 Department of Veterinary Medicine and Animal Sciences, University of Milan, 26900 Lodi, Italy

**Keywords:** optic lobe, serotonin, SERT, lamina, medulla, lobula, honeybee

## Abstract

**Simple Summary:**

Serotonin is ubiquitously expressed in vertebrates and invertebrates, where it regulates specific behavioural patterns. Though the specific effects of serotonin release in the optic lobe are not entirely known, increasing evidence associates the serotonergic system with optic lobe-mediated behaviours. In this study, the localization of serotonin transporter (SERT) was immunohistochemically analysed in the optic lobes of moderate, docile and aggressive worker honeybees. SERT-immunoreactive fibres were stratified in the optic lobe and distributed in the three visual neuropils: lamina, medulla and lobula. Interestingly, SERT immunoreactivity was inversely related to aggressive behaviour. The present study indicates that low levels of serotonin in the optic lobe are associated with aggressive behaviour.

**Abstract:**

Visual information is processed in the optic lobes, which consist of three retinotopic neuropils. These are the lamina, the medulla and the lobula. Biogenic amines play a crucial role in the control of insect responsiveness, and serotonin is clearly related to aggressiveness in invertebrates. Previous studies suggest that serotonin modulates aggression-related behaviours, possibly via alterations in optic lobe activity. The aim of this investigation was to immunohistochemically localize the distribution of serotonin transporter (SERT) in the optic lobe of moderate, docile and aggressive worker honeybees. SERT-immunoreactive fibres showed a wide distribution in the lamina, medulla and lobula; interestingly, the highest percentage of SERT immunoreactivity was observed across all the visual neuropils of the docile group. Although future research is needed to determine the relationship between the distribution of serotonin fibres in the honeybee brain and aggressive behaviours, our immunohistochemical study provides an anatomical basis supporting the role of serotonin in aggressive behaviour in the honeybee**.**

## 1. Introduction

The capacity of insects to receive sensory input, process information and respond with a specific behaviour is made possible by the brain [1]. The biogenic amine serotonin is widely distributed in the brain of the honeybee, where it modulates a great variety of behavioural processes, such as learning, memory and aggression [2,3]. Accordingly, in honeybees, differences in serotonin activity are related to specific behavioural patterns [4]. The serotonin transporter (SERT) plays a key role in the regulation of serotonin levels in the synapse. In fact, this plasma membrane transporter protein is responsible for the reuptake of serotonin into presynaptic neurons [5]. Interestingly, the serotonergic system, including SERT, is highly conserved between vertebrates and invertebrates [6,7].

The optic lobe, a complex structure located in the protocerebrum of honeybees, consists of three retinotopic neuropils. These are the lamina, the medulla and the lobula. Their function consists, before the information is integrated in the high-order brain centre, of a consecutive step aimed at visual conversion and segregation [8]. Interestingly, brain activity in the optic lobes precedes behavioural choices, suggesting that attention-like processes are pushed far out into the sensory periphery [9]. Immunohistochemical studies indicate that the honeybee optic lobe receives serotonergic fibres from the cell groups located in the protocerebrum [10]. Serotonin exerts its effects in the optic lobe, acting on 5-HT1A and, to some extent, 5-HT2 receptors [11,12]. Previous studies demonstrated that serotonin is involved in the regulation of honeybee phototactic behaviour, and the 5-HT1A receptor is the main mediator of this effect. In addition, serotonin in the optic lobe can modulate visual responses, such as the motion-sensitive visual antennal reflex and phototactic reflex [11,13]. The aforementioned studies support the idea that the interaction of the optic lobes and the serotoninergic system may play a primary role in the management of behaviours.

The goal of the present study was to determine the distribution of serotonin fibres in the optic lobes of different honeybees (docile, moderate and aggressive) using an immunofluorescence method with primary antibody against serotonin transporter (SERT). Although the presence of serotonin immunoreactive fibres has been investigated using primary antibody against serotonin [10,14], different studies reported that antibodies against SERT were preferable to determine serotonergic fibres [15]. Our results provide initial insights into the serotonergic modulation of aggression-related behaviours in honeybee.

## 2. Materials and Methods

### 2.1. Animals and Fixation

Thirty worker honeybees (ten bees in the moderate group, ten bees in the docile group and ten bees in the aggressive group) were used in this study.

In detail, the honeybees analysed in this study belonged to a genetic selection program where the docility of 108 colonies was assessed four times a year by an expert beekeeper, following Büchler et al., 2013 and Uzonov et al., 2015 [16,17]. The final phenotype of each colony was obtained from the averages from four tests carried out between March and June. Based on their mean phenotype, the ten best colonies (docility average: 3.43), the ten most aggressive colonies (docility average: 1.8) and ten colonies as a moderate group (docility average: 2.67) were sampled in October 2021. On the same day, one worker bee per colony was taken from the colony and killed by freezing prior to brain analysis.

Docility is composed of two traits: gentleness and calmness. Gentleness, also known as defensive behaviour, measures aggressiveness against humans, while calmness measures the stillness and immobility of worker bees on the comb during the inspection [16]. Both traits were evaluated together and scored on a scale from 1 to 4. A score of 1 referred to the most aggressive behaviour and indicated that the colony is nervous: many bees flew around the comb and tried to sting despite the use of smoke. A score of 2 meant that few bees left the comb and tried to sting, even if smoke was used. A score of 3 indicated that the bees moved on the comb without flying and it was possible to avoid stings with the use of smoke. A score of 4 referred to the calmest behaviour; bees stayed still on the comb, and it was not necessary to use smoke or protective clothes during their handling. Half-points (1.5, 2.5, 3.5) were used to discriminate colonies with intermediate behaviours. Docility is an important trait in beekeeping, as calm and gentle bees are easier to manage and there is less risk for beekeepers of being stung or injuring the queen during the inspection of the apiary. For this reason, docility is one of the most widely selected phenotypes in breeding programs, together with honey production, swarming tendency and varroa resistance [18]. Thanks to breeding selection, docility has significantly increased since 1990 [19].

The animals were deeply anesthetized with ice and perfused with a solution of 4% paraformaldehyde in 0.1 M sodium phosphate buffer, pH 7.4. The brains were dissected, fixed in the same fixative for 3 h and washed (4 × 15 min) in phosphate-buffered saline (PBS). The brains were then cryoprotected in 30% sucrose solution in PBS, pH 7.4, at +4 °C for 48 h. Tissues were dipped in Tissue Tek^®^ (Sakura Finetek Europe) mounting medium and stored at 4 °C overnight, then frozen in isopentane (Sigma-Aldrich, Co., 270342-1L, St. Louis, MO, USA) cooled in liquid nitrogen. Serial coronal sections (15 μm thickness) were cut on a cryostat and mounted on coverslips coated with poly-L-lysine (Thermo Scientific, MA, USA).

### 2.2. Immunostaining

The final concentrations of primary antibody were established by performing immunofluorescence experiments using different dilution patterns. First of all the cryosections were hydrated and washed in PBS (3 × 10 min). A solution containing 10% normal goat serum (Colorado Serum Co., Denver, CO, USA, #CS 0922) and 0.3% Triton X-100- in 0.02 M PBS (Sigma-Aldrich, Co., 9036-19-5, St. Lous, MO, USA) was used to block nonspecific binding through incubation of cryosections for 40 min at room temperatures (RT). Subsequently, after three 10-min washes in a solution containing 1% normal goat serum and 0.3% Triton X-100 in 0.02 M PBS, the cryosections were incubated overnight at 4 °C in the following primary antisera: rabbit anti-SERT polyclonal antibody (diluted 1:100, code 24330, ImmunoStar, WI, USA) dissolved in 1% normal goat serum (Colorado Serum Co., Denver, CO, USA, #CS 0922) and 0.3% Triton X-100 in 0.02 M PBS. After washing in PBS (3 × 10 min), the sections were incubated for 2 h at RT with the secondary antibody solution, which contained Alexa 594-conjugated goat anti-rabbit IgG (1:200, #A11012, Molecular Probes, Leiden, the Netherlands) diluted in 1% normal goat serum and 0.3% Triton X-100 in 0.02 M PBS. The sections were then washed with 0.02 M PBS and mounted in buffered glycerol at pH 8.6 with 4′,6-diamidino-2-phenylindole (DAPI) (Santa Cruz Biotechnology, Santa Cruz, CA, USA), a cell-permeable DNA-binding dye. The specificity of the rabbit polyclonal antibody directed against insect SERT was determined by the manufacturer. In the present experiments, the pre-adsorption test with SERT peptide control (code 24332, ImmunoStar, WI, USA) abolished the immunostaining (Supplementary File S1). In addition, control sections incubated without the primary antibody resulted in the complete disappearance of stained profiles. The omission, as well as the replacement of the secondary antibody with inappropriate secondary antibody, resulted in the elimination of all immunohistochemical staining.

### 2.3. Analysis of Sections

Four sections for each animal were examined using a Nikon H550L (Nikon Instruments, Natori, Japan) equipped with the appropriate filter cubes to differentiate the fluorochromes employed. The TRITC filter was employed for Alexa 594 (EX 540/25; DM 565; BA 605–655) and the DAPI/Hoechst/Alexa Fluor 350 filter for DAPI (EX 375/28; DM 415; BA 460/50). Bilateral observations of the optic lobes were carried out, proceeding rostrocaudally. Histological specimens were evaluated blindly. The images were recorded, with identical parameters for the three groups (moderate, docile and aggressive), by a Nikon DS-Qi1Mc digital camera (Nikon Instruments, Japan) and Nikon Elements software, version 4.10. Slight adjustments to the contrast and brightness of the figures were applied using Adobe Photoshop CS3 Extended 10.0 software (Adobe Systems, San Jose, CA, USA). The same adjustments were applied to the figures. The threshold technique in ImageJ (version IJ 1.46r, downloaded from http://imagej.nih.gov/ij/download.html accessed on 20 July 2019) was used to analyse the percentage of the image with SERT immunoreactivity. The images were first “thresholded” so that only the pixels above the threshold level were counted as positive labelling elements. The threshold level was set case by case with respect to the background level of the negative control sections. The pixel area occupied by SERT-positive elements above the threshold level was measured and the percentage fractions were calculated. The data were expressed as means ± standard deviation (SD) with a significance level of *p* < 0.05 and, after being tested for normality with a Shapiro–Wilk test, analysed using a non-parametric Kruskal–Wallis test. Subsequently, the differences between moderate, docile and aggressive groups were evaluated using the Wilcoxon test. The data analysed in this study are available in Supplementary File S1.

## 3. Results

### Distribution of SERT Immunoreactivity in the Optic Lobe

The optic lobe of the honeybees, as is already known, was found to be composed of three visual neuropils: the lamina, medulla and lobula. The lamina was most peripherally located in the optic lobe, just below the retina. The lamina showed two layers: a layer of cell bodies and a fibre layer. The next neuropil sheet, the medulla, was composed of different layers (containing somata and cellular processes), including the outer and inner medulla and the serpentine layer. The third neuropil region, the lobula complex, consisted of a single neuropil. The outer chiasma connected the lamina to the medulla, whereas the inner chiasma connected the medulla to the lobula.

**Figure 1:**
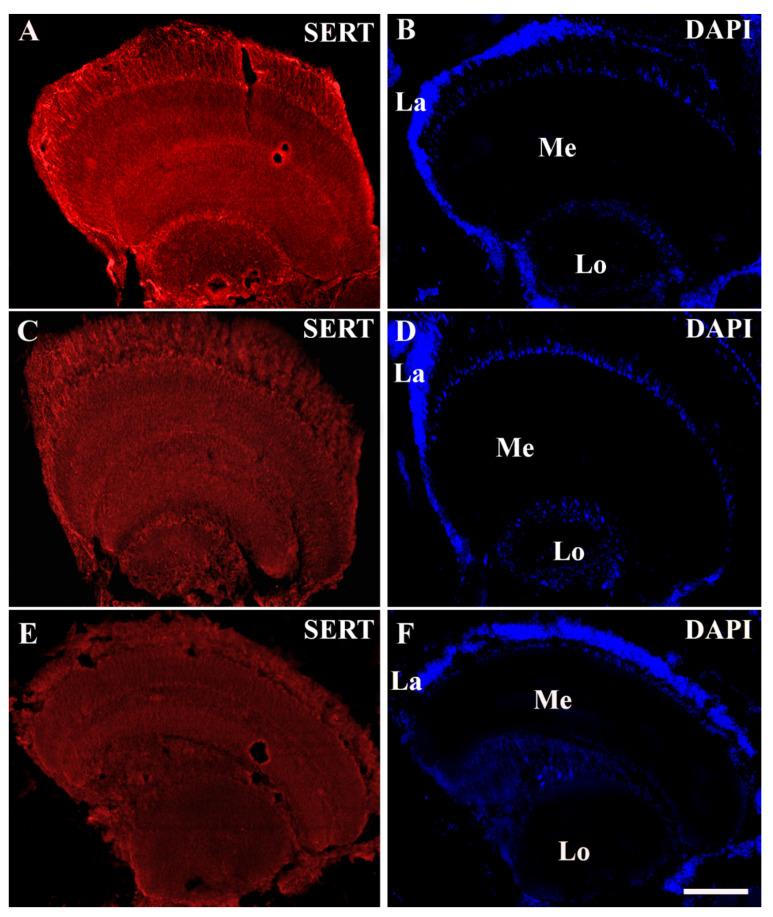
General appearance of serotonin transporter (SERT) immunoreactivity in the optic lobes of the docile (**A**,**B**), moderate (**C**,**D**) and aggressive (**E**,**F**) honeybees. Note that SERT immunoreactable 100 µm. Abbreviations: La, lamina; Me, medulla; Lo, lobula.

**Figure 2:**
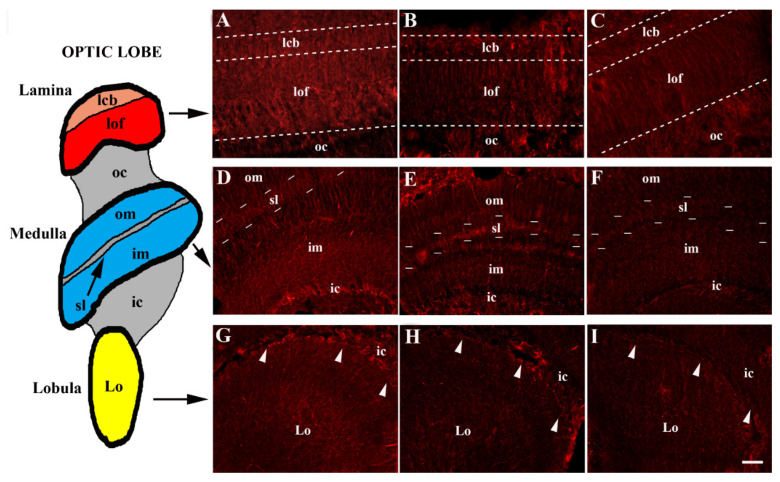
Distribution of serotonin transporter (SERT)-immunoreactive fibres in lamina (**A**–**C**), medulla (**D**–**F**) and lobula (**G**–**I**) of docile (**A**,**D**,**G**), moderate (**B**,**E**,**H**) and aggressive (**C**,**F**,**I**) honeybees. In the lamina, SERT-immunoreactive fibres were located in the cell body layer (lcb). The lamina showed many SERT-immunoreactive fibres in the layer of fibres (lof). The outer medulla (om), the serpentine layer (sl) and the inner medulla (im) contained many SERT-immunoreactive fibres distributed across the different strata of the lobula. Immunoreactive fibres were also located in the outer chiasma (oc) and inner chiasma (ic). Note that, in the lamina, medulla and lobula, the immunoreactivity for the SERT decreased from docile to aggressive honeybees. Arrowheads indicate the border between lobula and inner chiasma. Scale bar: 10 µm.

The honeybee optic lobe was innervated with SERT-immunoreactive fibres and demonstrated a specific pattern of innervation in the three visual neuropils: the lamina, medulla and lobula (Figure 1 and Figure 2). In the lamina, serotoninergic fibres were located in the cell body layer, where the somata of unipolar and multipolar neurons are located. The lamina exhibited many SERT-immunoreactive fibres in the layer of the fibres, which was a stratum close to the outer chiasma (Figure 2A–C).

In the medulla, serotoninergic fibres were present mainly in the serpentine layer and adjacent neuropils of the outer and inner medulla (Figure 2D–F). Immunoreactive fibres were distributed in the different strata of the lobula (Figure 2G–I). Immunoreactive fibres could be observed in the outer and inner chiasmata (Figure 2).

**Figure 3:**
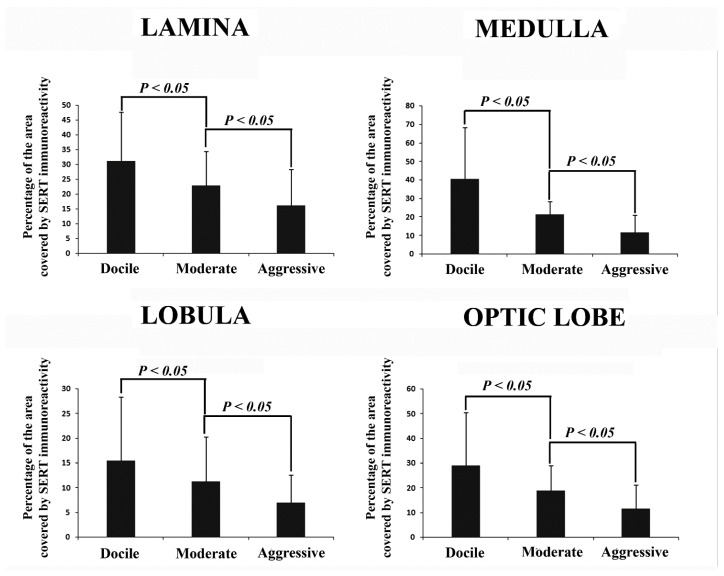
Histograms showing the mean percentages of the image covered by SERT immunoreactivity ± standard deviation (SD) in the lamina, medulla and lobula and in the optic lobe as a complex for docile, moderate and aggressive honeybees.

The distribution of SERT immunoreactivity varied in the three visual neuropils (Figure 1, Figure 2 and Figure 3). In the optic lobe of all the animal groups (moderate, docile, aggressive), the lobula was found to have the lowest percentage covered by SERT immunoreactivity (*p* < 0.05). In moderate honeybees, the percentage of the lamina covered by SERT-immunoreactivity was very similar to that in the medulla, whereas in docile honeybees, the greatest immunoreactivity was calculated in the medulla (*p* < 0.05). In contrast, in the aggressive group, the percentage covered by SERT immunoreactivity was higher in the lamina than in the medulla (*p* < 0.05). 

A significant variation in SERT immunoreactivity was observed among optic lobes within the different honeybee groups (moderate, docile and aggressive). The lowest SERT immunoreactivity was observed in the aggressive group. In particular, a remarkable decrease in SERT-immunoreactive stain density was seen in all the visual neuropils of the aggressive honeybees (Figure 1, Figure 2 and Figure 3).

## 4. Discussion

The optic lobe is a complex extension of the brain and represents the processing centre for visual information. It is made up of several parts, among which are the lamina (distal), the outer chiasma, the medulla, the inner chiasma and the lobula (proximal) [8,9]. In addition, the optic lobe of honeybees also regulates attention-like processes [9]. In the present work, we reported for the first time the distribution of SERT immunoreactivity in the optic lobe of honeybees. Previous studies have demonstrated the presence of serotonergic fibres in the honeybee brain using primary antibodies against serotonin [10,14]. To evaluate the density of serotonergic fibres, we preferred to use primary antibody against SERT. In fact, previous research reported that antibodies against SERT were preferable to determine serotonergic fibres, because, unlike serotonin, SERT is less liable to metabolism and, for this reason, a more stable marker to stain serotonergic fibres [15]. The stratified pattern of distribution of serotonergic fibres in the optic lobe observed in our study was similar to that observed previously using different methods [10]. However, the antibody directed against SERT labelled many fibres located in the outer and inner medulla.

Biogenic amines play a crucial role in the control of insect responsiveness. Serotonin has been repeatedly related to aggressiveness in invertebrates [20], and serotonin is a component of aggressive behaviours [21,22]; however, the potential role of serotonin in aversive responsiveness has not been addressed until now. The presence of serotoninergic fibres in the optic lobe seems to indicate their involvements in visual information processing. Accordingly, serotonin acts as a down-regulator of sting responsiveness and increases non-specific responsiveness [23]. In addition, injection of serotonin into the optic lobe inhibits the response to moving stripe patterns and decreases the amplitude of field potentials [24].

Though the specific effects of serotonin release in the optic lobe are not completely understood, our results link the serotonergic system with optic lobe-mediated behaviours. In particular, we observed that the highest and the lowest SERT immunoreactivity were located in the docile and aggressive groups, respectively. These data suggest that low levels of serotonin in the optic lobe are associated with aggressive behaviour. Interestingly, the distribution of SERT immunoreactivity varied among the three visual neuropils with the behaviour, suggesting a specific local function of serotonin in each visual neuropil.

In the optic lobe, the serotonin release determines particular effects depending on which neuron is engaged, the different kinds of synapsis formed and the classes of receptors activated [6,7]. Therefore, in order to provide the basis for subsequent evaluations of the functional role of serotonin in the honeybee optic lobe, an accurate description of serotonin fibres’ ultrastructure and receptor distribution is necessary.

## 5. Conclusions

The description of the serotonergic innervation of the optic lobe provides information for studies that seek to understand the mechanism by which serotonin modulates aggression-related behaviours through its activity in different brain regions. Though more information is needed to evaluate the functional significance of the serotonin innervation of the optic lobe, such as studies involving SERT knock-out animals, the present study demonstrates that serotonin may influence different components of optic lobe function, including aggression-related behaviours.

## Data Availability

Data are available in Supplementary File S1.

## Supplementary Materials

The following supporting information can be downloaded at: https://www.mdpi.com/article/10.3390/ani12162032/s1, Supplementary File S1: Percentage of the image covered by SERT immunoreactivity.

## References

[1] Ito K., Shinomiya K., Ito M., Armstrong J.D., Boyan G., Hartenstein V., Harzsch S., Heisenberg M., Homberg U., Jenett A. (2014). A systematic nomenclature for the insect brain. Neuron.

[2] Dierick H.A., Greenspan R.J. (2007). Serotonin and neuropeptide F have opposite modulatory effects on fly aggression. Nat. Genet..

[3] Sitaraman D., Zars M., LaFerriere H., Chen Y.-C., Sable-Smith A., Kitamoto T., Rottinghaus G.E., Zars T. (2008). Serotonin is necessary for place memory in drosophila. Proc. Natl. Acad. Sci. USA.

[4] Scheiner R., Baumann A., Blenau W. (2006). Aminergic control and modulation of honeybee behaviour. Curr. Neuropharmacol..

[5] Martin C.A., Krantz D.E. (2014). Drosophila melanogaster as a genetic model system to study neurotransmitter transporters. Neurochem. Int..

[6] Tierney A.J. (2018). Invertebrate serotonin receptors: A molecular perspective on classification and pharmacology. J. Exp. Biol..

[7] Moutkine I., Collins E.L., Béchade C., Maroteaux L. (2019). Evolutionary considerations on 5-HT2 receptors. Pharmacol. Res..

[8] Brandt R., Rohlfing T., Rybak J., Krofczik S., Maye A., Westerhoff M., Hege H.-C., Menzel R. (2005). Three-dimensional average-shape atlas of the honeybee brain and its applications. J. Comp. Neurol..

[9] Paulk A.C., Stacey J.A., Pearson T.W.J., Taylor G.J., Moore R.J.D., Srinivasan M.V., van Swinderen B. (2014). Selective attention in the honeybee optic lobes precedes behavioral choices. Proc. Natl. Acad. Sci. USA.

[10] Schürmann F.W., Klemm N. (1984). Serotonin-immunoreactive neurons in the brain of the honeybee. J. Comp. Neurol..

[11] Thamm M., Balfanz S., Scheiner R., Baumann A., Blenau W. (2010). Characterization of the 5-HT1A receptor of the honeybee (*Apis mellifera*) and involvement of serotonin in phototactic behavior. Cell. Mol. Life Sci..

[12] Thamm M., Rolke D., Jordan N., Balfanz S., Schiffer C., Baumann A., Blenau W. (2013). Function and distribution of 5-HT2 receptors in the honeybee (*Apis mellifera*). PLoS ONE.

[13] Erber J., Kloppenburg P. (1995). The modulatory effects of serotonin and octopamine in the visual system of the honey bee (*Apis mellifera* L.). J. Comp. Physiol. A.

[14] Seidel C., Bicker G. (1996). The developmental expression of serotonin-immunoreactivity in the brain of the pupal honeybee. Tissue Cell.

[15] Nielsen K., Brask D., Knudsen G.M., Aznar S. (2006). Immunodetection of the serotonin transporter protein is a more valid marker for serotonergic fibers than serotonin. Synapse.

[16] Buchler R., Andonov S., Bienefeld K., Costa C., Hatjina F., Kezic N., Kryger P., Pivak M., Uzunov A., Wilde J. (2013). Standard methods for rearing and selection of Apis mellifera queens. J. Apic. Res..

[17] Uzunov A., Büchler R., Bienefeld K. (2015). Performance Testing Protocol. A Guide for European Honey Bee Breeders.

[18] Uzunov A., Brascamp E.W., Buchler R. (2017). The basic concept of honey bee breeding programs. Bee World.

[19] Hoppe A., Du M., Bernstein R., Tiesler F.K., Kärcher M., Bienefeld K. (2020). Substantial genetic progress in the international Apis mellifera carnica population since the implementation of genetic evaluation. Insects.

[20] Kravitz E.A., Huber R. (2003). Aggression in invertebrates. Curr. Opin. Neurobiol..

[21] Bubak A.N., Watt M.J., Yaeger J.D.W., Renner K.J., Swallow J.G. (2020). The stalk-eyed fly as a model for aggression—Is there a conserved role for 5-HT between vertebrates and invertebrates?. J. Exp. Biol..

[22] Poetini M.R., Musachio E.A.S., Araujo S.M., Almeida F.P., Dahleh M.M.M., Bortolotto V.C., Janner D.E., Pinheiro F.C., Ramborger B.P., Roehrs R. (2021). Iron overload during the embryonic period develops hyperactive like behavior and dysregulation of biogenic amines in drosophila melanogaster. Dev. Biol..

[23] Tedjakumala S.R., Aimable M., Giurfa M. (2014). Pharmacological modulation of aversive responsiveness in honey bees. Front. Behav. Neurosci..

[24] Birmingham J.T., Tauck D.L. (2003). Neuromodulation in invertebrate sensory systems: From biophysics to behavior. J. Exp. Biol..

